# Disease‐modifying therapies and T1 hypointense lesions in patients with multiple sclerosis: A systematic review and meta‐analysis

**DOI:** 10.1111/cns.13815

**Published:** 2022-02-25

**Authors:** Amir Valizadeh, Mohammad Reza Fattahi, Maryam Sadeghi, Mehrnush Saghab Torbati, Mohammad Ali Sahraian, Amir Reza Azimi

**Affiliations:** ^1^ Neuroscience Institute Tehran University of Medical Sciences Tehran Iran; ^2^ Islamic Azad University of Zahedan Zahedan Iran; ^3^ Multiple Sclerosis Research Center Neuroscience Institute Tehran University of Medical Sciences Tehran Iran

**Keywords:** alemtuzumab, cladribine, dimethyl fumarate, fingolimod, glatiramer acetate, interferon beta‐1a, magnetic resonance imaging, multiple sclerosis

## Abstract

**Background:**

Previous research has shown that cerebral T1 hypointense lesions are positively correlated with the disability of multiple sclerosis (MS) patients. Hence, they could be used as an objective marker for evaluating the progression of the disease. Up to this date, there has not been a systematic evaluation of the effects of disease‐modifying therapies (DMTs) on this prognostic marker.

**Objectives:**

To evaluate the effects of FDA‐approved DMTs on the numbers and volume of T1 hypointense lesions in adult patients with MS.

**Methods:**

We included studies with the mentioned desired outcomes. In March 2021, we searched MEDLINE (Ovid), Embase, and CENTRAL to find relevant studies. All included studies were assessed for the risk of bias using the RoB‐2 tool. Extracted data were analyzed using a random‐effects model. Certainty of evidence was assessed using GRADE.

**Results:**

Thirteen studies with 7484 participants were included. Meta‐analysis revealed the mean difference between the intervention and comparator groups for the number of lesions was −1.3 (95% CI: −2.1, −0.5) and for the mean volume of lesions was −363.1 (95% CI: −611.6, −114.6). Certainty of evidence was judged to be moderate. Heterogeneity was considerable.

**Discussion:**

DMTs reduce the number and volume of T1 hypointense lesions. Although, these findings must be interpreted cautiously due to the high values of heterogeneity.

## Graphical abstract

Objectives: To evaluate the effects of FDA‐approved disease‐modifying therapies (DMTs) on the numbers and volume of T1 hypointense lesions in adult patients with multiple sclerosis (MS). Results: Thirteen studies with 7484 participants were included. Mean difference for the number of lesions was −1.3 (−2.1, −0.5). Mean difference for the volume of lesions was −363 (−611, −114). Heterogeneity was considerable. Certainty of evidence was moderate. Conclusion: DMTs reduce the number and volume of T1 hypointense lesions. Although, these findings must be interpreted cautiously due to the high values of heterogeneity.

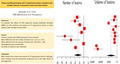

## 1 INTRODUCTION

Multiple sclerosis (MS) is a complex neurological disease that is characterized by inflammation, demyelination, gliosis, and neuronal loss. Based on the disease course, it groups into seven categories, of which relapsing‐remitting MS is the most common.^1^ Clinical symptoms typically first develop in young adults. Gradually, a progressive course then ensues with permanent disability in 10 to 15 years. Neuroimaging studies have shown functional, structural, and microstructural alterations in the central nervous system of the patients.^2^, ^3^, ^5^ Its pathology is believed to be immune‐mediated, incorporating several immune and neurodegenerative processes.

Disease‐modifying therapies (DMTs) are a medication class that modulate, modify, or suppress the immune system and are used to treat patients with MS.^5^ Interferon beta‐1a (a drug in the DMT class) was the first medication that was found to alter the course of the disease in a controlled study of MS and was subsequently approved by the FDA.^6^ This class consists of many different drugs that affect MS patients via different mechanisms, and there are still more of them in development. Fifteen medications of this class have been recently listed in the Essential Medicines List by the World Health Organization.^7^ These medications are believed to significantly affect the disability in MS patients. A 15‐year cohort has recently confirmed this and concluded that DMTs are effective in improving disability outcomes in patients in the long term.^8^ Another recent study that followed 216 stable MS patients after they discontinued DMTs for about 4.6 years found that patients experienced a considerable progression of disability, regardless of their age and sex.^9^ Given these findings and the frequent use of DMTs in clinical practice, it is essential that their effects on various health outcomes be well understood to provide the foundation for evidence‐based clinical practices. One of the outcomes of interest could be magnetic resonance imaging (MRI) metrics. MRI is a sensitive paraclinical test for diagnosis and assessment of disease progression in MS and is often used to evaluate therapeutic efficacy. Various types of lesions could be identified using MRI. One of the most important types of lesions is white matter lesions that look hypointense on T1‐weighted images.

T1 hypointense lesions are believed to correspond to axonal loss, white matter destruction, axonal loss, and irreversible clinical outcome.^10^ On the contrary, white matter lesions on T2‐weighted images are believed to correspond to a variety of different histopathological changes such as edema, inflammation, demyelination, gliosis, and axonal loss.^11^ These indicate that T1 hypointense lesions might be a more specific MRI metric to evaluate disease activity in MS patients. Furthermore, our previous meta‐analysis revealed that there might be a mild‐to‐moderate correlation between the mean volume of T1 hypointense lesions and measures of clinical disability.^12^

Given the importance of T1 hypointense lesions and the importance of DMTs in the treatment of MS, this systematic review aims to evaluate the efficacy of DMTs on the numbers and volume of T1 hypointense lesions in MS patients to present an explicit summary of the findings up to this date.

## 2 METHODS

Design and methods used for this review comply with Centre for Reviews and Dissemination (CRD’s) Guidance For Undertaking Reviews in Healthcare^13^ and are reported in line with Preferred Reporting Items for Systematic Reviews and Meta‐Analyses 2020.^14^

### 2.1 Eligibility criteria

**(P) Population:** Adult patients diagnosed with any phenotype of MS based on the McDonald criteria^15^ or Definite MS based on the Poser criteria.^16^

(I) Index: FDA‐approved DMTs, at any dose, frequency, or administration route. Concomitant interventions are allowed if they were used equally in all intervention groups in the trial. These include: alemtuzumab, beta‐1a interferon, beta‐1a peginterferon, cladribine, daclizumab, dimethyl fumarate, fingolimod, glatiramer acetate, mitoxantrone, natalizumab, ocrelizumab, ozanimod, rituximab, siponimod, and teriflunomide.

(C) Comparator: placebo, routine care, or no treatment regimen.

(O) Outcome: number and mean volume change of T1 hypointense lesions on cerebral MRI.

(T) Timing: any.

(S) Setting: any.

### 2.2 Information sources and search strategy

The search employed sensitive topic‐based strategies designed for each database with no time frame, language, or geographical restrictions. We performed our search on the 20th of March, 2021.

Databases:

● MEDLINE (Ovid)
● Embase
● Cochrane Central Register of Controlled Trials

We also examined the forward and backward citations of the included studies using Scopus.

Our search strategies are presented in Appendix A. Our search included highly sensitive search filters for clinical trials from the InterTASC Information Specialists’ Sub‐Group (ISSG)^17^ and Cochrane Collaboration.^17^

### 2.3 Selection process

MaS and MT independently screened records. Then, full texts of potentially eligible studies were retrieved. A study was included when both reviewers independently assess it as satisfying the inclusion criteria. AV was consulted in cases of disagreement.

### 2.4 Data collection process and data items

Using a standardized form, MaS and MT extracted the data independently. Disagreements were resolved through discussion. In cases of missing data, original authors were tried to be reached.

The main outcomes of interest were the mean difference (MD) of T1 hypointense lesion numbers and mean volume change from the baseline following receiving DMTs between the intervention and comparator groups. Other variables included the following: sample characteristics, sample size, study methods, inclusion and exclusion criteria, MRI settings used, founding sources, and declarations of interests.

### 2.5 Study risk of bias assessment

MaS and MT assessed the risk of bias for each included study. Disagreements were resolved through discussion. We used the Revised Cochrane risk of bias tool for randomized trials (RoB‐2).^18^ The tool, alongside the conditions to meet the answer “yes” for each signaling question in our review, is presented in Appendix B. This tool consists of five domains: bias arising from the randomization process, bias due to deviations from the intended interventions, bias due to missing outcome data, bias in the measurement of the outcome, and bias in the selection of the reported results.

### 2.6 Synthesis methods

#### 2.6.1 Preparing for synthesis

Studies that met our eligibility criteria and reported our outcomes of interest were assessed to be eligible for quantitative synthesis. We expected our outcomes of interest to be reported as the change from baseline as numbers and means (μ) after receiving the intervention for both the intervention and comparator group. Because these outcomes are expected to be reported in the same unit (numbers and millimeters), we will use mean differences (MDs) for the statistical analysis. We used R version 4 “dmetar”^19^ and “meta”^20^ packages as the software for our data synthesis.

We planned to present the results of each included study (with its 95% confidence interval) in conjunction with the synthesized effect estimate for each main outcome in a separate forest plot.

#### 2.6.2 Statistical synthesis methods

We calculated the variance and standard error of the extracted MDs. Because of the nature of our interventions of interest, we expected some variability in the studies. Thus, we performed meta‐analyses on those values using the random‐effects model.

#### 2.6.3 Methods to explore heterogeneity

We inspected our data visually to investigate the possibility of statistical heterogeneity. We also calculated χ^2^ and I^2^ statistics. *χ* ^2^ statistics are considered to be substantial if either τ^2^ was greater than zero, or there was a *p*‐value of <0.10. I^2^ statistics quantify inconsistency across studies to assess the impact of heterogeneity on the meta‐analysis.^21^ *I* ^2^ statistics were interpreted as follows:

● 0%–40%: might not be important;
● 30%–60%: moderate heterogeneity;
● 50%–90%: substantial heterogeneity;
● 75%–100%: considerable heterogeneity.

To investigate the possible sources of heterogeneity, subgroup analyses were conducted if at least 5 studies were available for each DMT.

#### 2.6.4 Sensitivity analyses

We analyzed the effects of excluding trials that were judged to be at high risk of bias across one or more of the “risk of bias” domains.

### 2.7 Certainty assessment

The strength of the overall body of evidence was assessed using the Grading of Recommendations, Assessment, Development and Evaluation (GRADE) framework,^22^ which takes into account eight criteria: risk of bias, consistency of effect, imprecision, indirectness, publication bias, large effect, dose–response, and plausible confounding. We did not assess publication bias and dose–response domains for our review due to the heterogeneous nature of our interventions of interest. AV rated the certainty of the evidence for the outcomes as “high,” “moderate,” “low,” or “very low.”

## 3 RESULTS

### 3.1 Study selection

**FIGURE 1:**
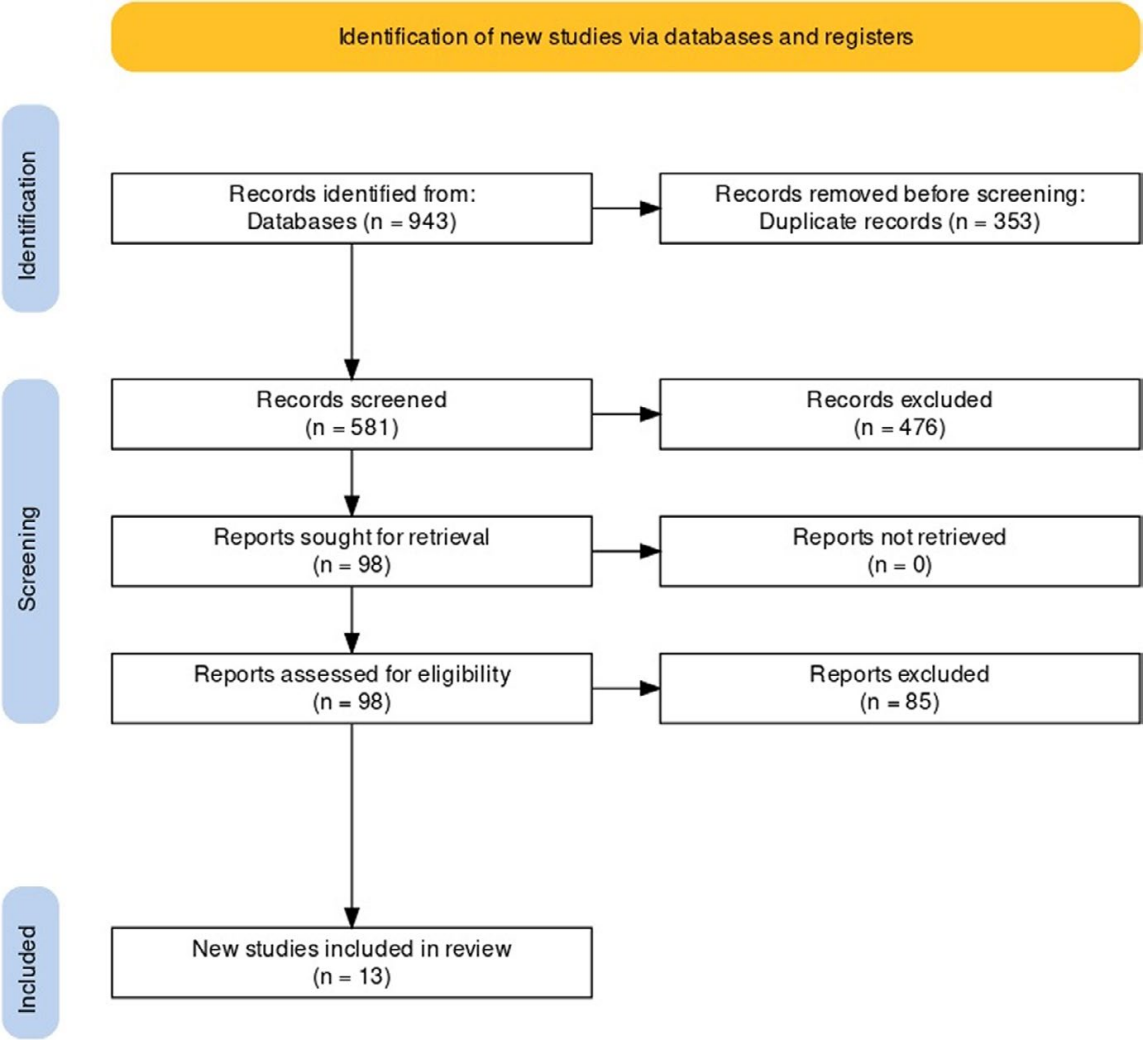
PRISMA Flow diagram of the study

For a detailed summary of the flow of studies, see the PRISMA flow diagram presented in Figure 1.

For this review, we identified 934 records in our primary search (454 CENTRAL, 351 Embase, and 119 MEDLINE (Ovid)). After removing duplicates, we screened the titles and abstracts of 581 records. Another 476 records were excluded at that stage, and 98 records remained for full‐text assessment. We excluded 85 studies that did not meet the eligibility criteria for our review, after assessing the full text of the records. In the end, we included 13 studies in this review.

### 3.2 Study characteristics

We included 13 studies^24^, ^25^, ^26^, ^27^, ^28^, ^29^, ^30^, ^31^, ^32^, ^33^, ^34^, ^35^, ^36^ with 7484 participants. Three studies only reported outcomes for the number of lesions,^26^, ^30^, ^32^ while five studies only reported outcomes for the mean volume change of lesions.^27^, ^29^, ^34^, ^35^, ^36^ The rest of the included studies reported results for both outcomes. One study, which contributed to both outcomes, reported results for comparing two different FDA‐approved DMT drugs in two different intervention groups against the comparator group.^30^ For a detailed summary of the characteristics of the included studies, see Table 1.

**TABLE 1:** Summary of the characteristics of the contributing studies

Study ID	Intervention	Dosage	Length of follow up (weeks)	Sample size (F/M)		Age (SD)	
				Intervention	Control	Intervention	Control
Arnold 2014	DMF	240mg bid, 240mg tid	96	276/84	141/39	38.4 (8.9)	38.3 (9.2)
Arnold 2017	P‐IFN beta−1a	125mcg q2w, 125mcg q4w	96	556/239	282/109	36	37
Brex 2001	IFN beta−1a	8mIU q2d	96	34/25	27/27	40.7 (8.4)	39.9 (8.0)
Comi 2001	GA	20mg qd	36	119	120	34.1 (7.4)	34.0 (7.5)
Comi 2017	IFN beta−1a	44mcg 2qw, 44mcg qw	240	230	102	‐	‐
Filippi 2000	Cladribine	0.7mg, 2.1mg	48	53	54	‐	‐
Kappos 2008	DMF	120mg qd, 120mg tid, 240mg tid	24	128/63	35/30	36.1 (9.6)	35.6 (8.2)
Miller 2015	DMF	240mg bid, 240mg tid	96	239/100	116/51	38.3 (9.4)	36.6 (9.1)
Miller 2015	GA	20mg qd	96	123/52	116/51	36.8 (8.8)	36.6 (9.1)
Nagtegaal 2014	IFN beta−1a	250mcg q2d	240	190/77	118/50	30.8 (7.6)	30.6 (7.2)
Radue 2010	Natalizumab	300mg q4w	116	442/147	420/162	–	–
Radue 2012	Fingolimod	0.5mg qd, 1.25mg qd	96	594/266	298/120	37 (8.8)	37.2 (8.6)
Wolinsky 2013	Teriflunomide	7mg qd, 14mg qd	108	510/215	275/88	38.25	39
Zivadinov 2007	IFN beta−1a	qw	144	17/11	19/7	45.0 (8.5)	47.0 (8.1)

Abbreviations: 2qw, twice a week; bid, twice a day; DMF, dimethyl fumarate; GA, glatiramer acetate; IFN, interferon; P‐INF, peginterferon; q2d, once every two days; qd, once a day; qw, once a week; tid, three times a day.

### 3.3 Risk of bias in studies

**FIGURE 2:**
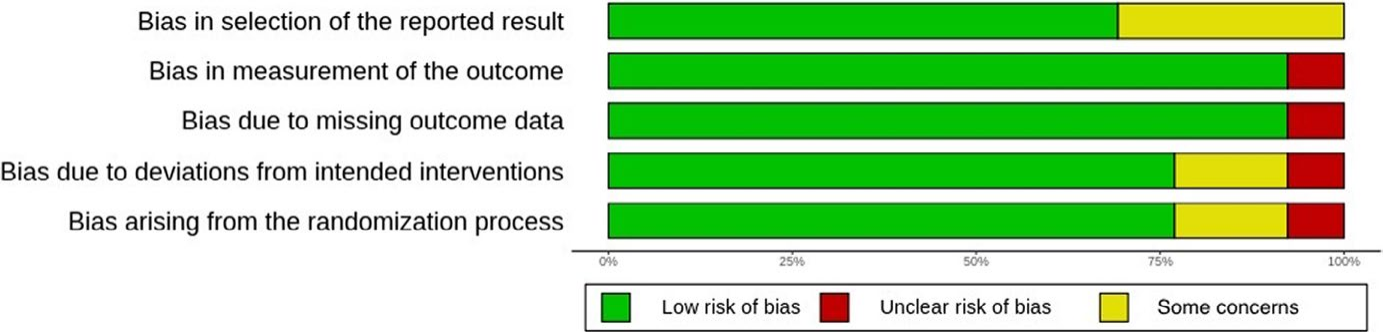
Risk of bias graph: review authors’ judgments about each risk of bias item presented as percentages across all included studies

**FIGURE 3:**
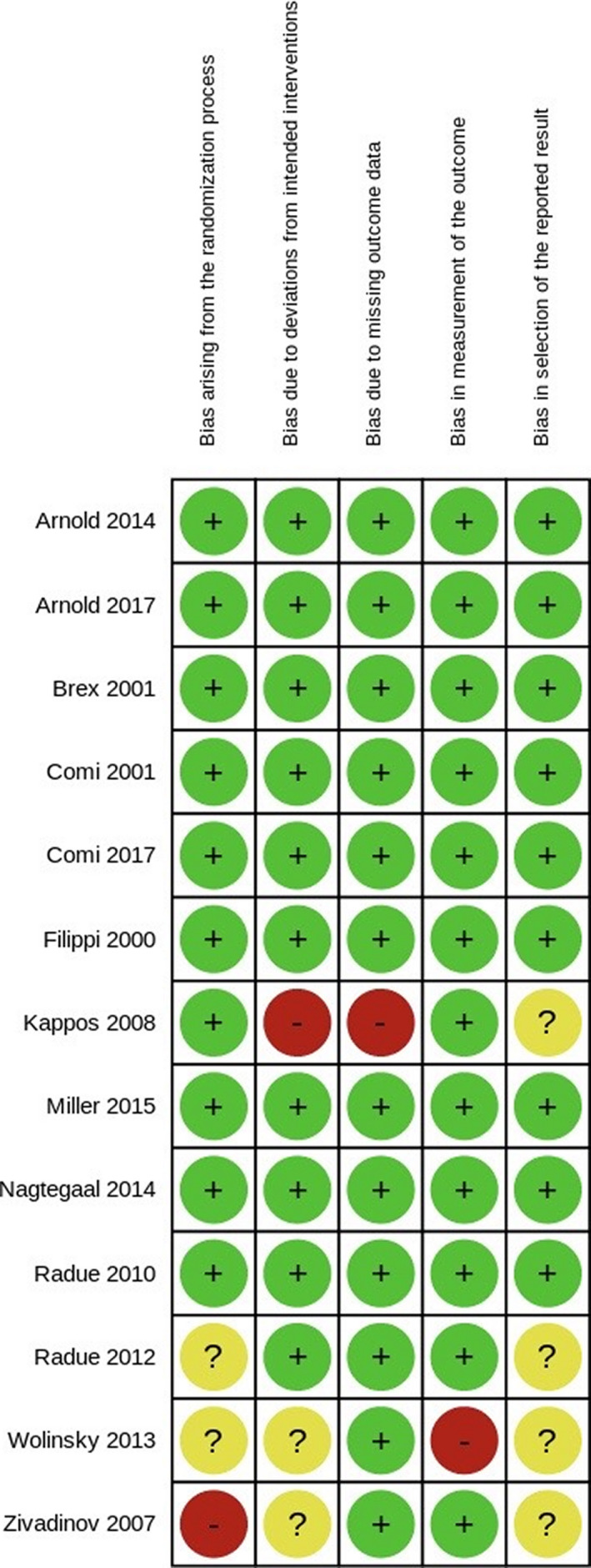
Risk of bias summary: review authors’ judgments about each risk of bias item for each included study

Figure 2 shows the risk of bias judgments for each domain in all included studies. Judgments for each domain across studies are shown in Figure 3.

One study was judged to be at high risk for bias arising from the randomization process,^35^ while two studies were at unclear risk.^34^, ^35^ One study was judged to be at high risk for bias due to deviations from the intended interventions,^30^, ^34^ while two studies were at unclear risk.^35^, ^36^ Only one study^30^, ^34^, ^35^ was judged to be at high risk for bias due to missing outcome data. Only one study was judged to be at high risk for bias in the measurement of the outcome.^34^ Four studies were judged to be at unclear risk for bias in the selection of the reported results.^30^, ^34^, ^35^, ^36^ No study seemed to be at high risk of bias for this domain.

Overall, three studies were judged to be at high risk of bias.^30^, ^35^, ^36^ These included one of the studies that contributed to syntheses for the number of lesions and two studies that contributed to syntheses for the mean volume change of lesions. There were some concerns regarding potential bias with one study,^33^ which contributed to syntheses for the mean volume change of lesions. The rest of the included studies were judged to be at low risk of bias.

### 3.4 Results of individual studies

**FIGURE 4:**
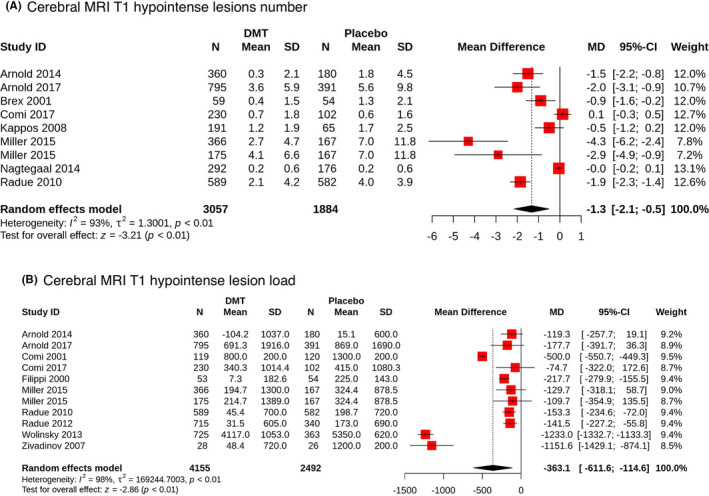
Forest plots of the overall synthesis: (A) The mean difference (MD) of cerebral MRI T1 hypointense lesions number between both groups. (B) The mean difference (MD) of cerebral MRI T1 hypointense lesion load between both groups

For both outcomes, the results of most individual studies were in the same direction (decreased number and volume of lesions). For the number of lesions, two studies^30^, ^32^ failed to detect a statistically significant difference between the groups (interventions were dimethyl fumarate and interferon beta‐1a, respectively), while one study^27^ found that their intervention (interferon beta‐1a) might actually increase the number of lesions (although it was not statistically significant either). For the volume of lesions, though all studies were in the same direction, five studies^24^, ^25^, ^28^, ^31^ failed to detect a statistically significant difference between the groups (interventions were dimethyl fumarate, peginterferon beta‐1a, interferon beta‐1a, dimethyl fumarate, and glatiramer acetate, respectively). For a detailed summary of the results of individual studies, check out the forest plots in Figure 4.

### 3.5 Results of syntheses

#### 3.5.1 Characteristics of contributing studies

A summary of the characteristics of the contributing studies is provided in Table 1.

##### Sample sizes

The median sample size was 388.5 participants (interquartile range 239–1088). The smallest sample size was 54,^35^ and the largest was 1278.^33^

##### Interventions

Four studies used interferon beta‐1a,^26^, ^28^, ^32^, ^36^ three used dimethyl fumarate,^25^, ^30^, ^31^ two used glatiramer acetate,^27^, ^31^ one used peginterferon beta‐1a,^23^ one used cladribine,^28^ one used natalizumab,^32^ one used fingolimod,^33^ and one used teriflunomide.^34^ It should be noticed that the study of Miller 2015^30^ had two interventional arms, dimethyl fumarate and glatiramer acetate.

##### Participants

Age of most of the participants in the included studies was in the range of 30–55 years. Although all studies were conducted on adults, three^28^, ^29^, ^33^ did not report the mean age of participants. Also, most studies matched the control group with the intervention group for sex. Only two studies failed to achieve this,^30^, ^36^ while three did not report the gender of the participants.^27^, ^28^, ^29^

##### Length of follow‐up

The median and mode of the length of follow‐up were both 96 weeks. The minimum was 24 weeks,^29^ and the maximum was 240 weeks.^28^, ^32^ Only three studies followed patients for less than 96 weeks.^27^, ^29^, ^30^

#### 3.5.2 Results of statistical syntheses

Thirteen studies obtained data sufficient for quantitative synthesis. Results of the meta‐analyses are presented in Figure 4.

For the lesions number outcome, the pooled sample size was 3057 in the intervention group and 1884 in the placebo group. Overall effect size (MD) was −1.3 (95% CI: −2.1, −0.5; *p*<0.01). This interprets as DMTs significantly decrease the number of T1 hypointense lesions in patients. For the lesion load outcome, the pooled sample size was 4155 in the intervention group and 2492 in the placebo group. Overall effect size (MD) was −363.1 (95% CI: −611.6, −114.6; *p *< 0.01). This interprets as DMTs significantly decrease the volume of T1 hypointense lesions in patients.

#### 3.5.3 Investigations of heterogeneity

For the number of lesions outcome, τ^2^ was 1.3 with a *p *< 0.01. I^2^ was 93%. For the volume of lesions outcome, τ^2^ was 169244 with a *p *< 0.01. I^2^ was 98%. These indicate there was considerable heterogeneity between the studies for both outcomes. To some extent, it was expected, as interventions differed between studies. We planned to perform subgroup analyses for each drug to evaluate the effect size specific for it, but, unfortunately, there were not enough studies for this purpose.

#### 3.5.4 Results of sensitivity analyses

We performed sensitivity analyses on studies that were judged to be at low risk of bias in all domains.

Sensitivity analysis for the number of lesions outcome included seven studies.^24^, ^25^, ^26^, ^28^, ^33^ The results of this synthesis were not much different from our overall results (n = 2866 intervention, 1819 placebo; MD = −1.5; 95% CI −2.4, −0.5; *p*<0.01).

Sensitivity analysis for the volume of lesions outcome included seven studies.^24^, ^25^, ^27^, ^28^, ^29^, ^31^, ^33^ The results of this synthesis were not much different from our overall results (*n* = 2687 intervention, 1763 placebo; MD = −203.5; 95% CI −313.8, −93.3; *p *< 0.01).

### 3.6 Certainty of evidence

For the “risk of bias” domain, four studies^30^, ^34^, ^35^, ^36^ were at risk of bias in at least two different domains, while three of them^30^, ^35^, ^36^ were at risk of bias in at least three different domains. Overall, we considered that included studies do not suffer from serious risks of bias. Thus, the certainty of the evidence was not downgraded. For the “consistency of the effect” domain, results of heterogeneity analyses indicated considerable inconsistencies between the included studies. Although such heterogeneity usually results in downgrading the certainty of evidence by two levels, but considering that the effect sizes of all studies for both outcomes were almost in the same direction (though in considerably different values), we decided to downgrade certainty of evidence by just one level. For the “imprecision” domain, the pooled sample size was 3057 in the intervention group and 1884 in the placebo group for the lesions number outcome, and for the lesion load outcome, it was 4155 and 2492, respectively. These sample sizes seem quite adequate. 95% CIs for the pooled effect sizes in our meta‐analyses, though wide, were on the same side of the plots. In conclusion, we believe there was no reason for downgrading the certainty of evidence regarding this domain. For the “indirectness” domain, population, interventions, and outcome measures of interest in all studies were the same as the ones intended for the review. Thus, the certainty of the evidence was not downgraded. For the “large effect” domain, though effect sizes for both outcomes were statistically significant, we believe upgrading the certainty of the evidence is not required. For the “plausible confounding” domain, we believe there were no plausible confounding factors in the studies to justify upgrading the certainty of evidence.

Overall, we downgraded the certainty of evidence by one level due to inconsistency between the results of the included studies. Thus, the overall certainty of the evidence is judged to be moderate.

## 4 DISCUSSION

### 4.1 Interpretation

To our knowledge, this is the first study that has systematically evaluated the effect of DMTs on the numbers and volume of T1 hypointense lesions in MS patients. Our analyses indicate, with moderate certainty, that the use of DMTs for MS patients results in decrease in the frequency (−1.3; 95% CI: −2.1, −0.5; *p *< 0.01) and volume (−363.1; 95% CI: −611.6, −114.6; *p *< 0.01) of these lesions, relative to placebo. Sensitivity analysis confirmed the robustness of our results against possible sources of bias. For a summary of main findings, see Table 2.

**TABLE 2:** Summary of the main findings

Outcome	Number of participants		Number of studies	Mean difference (95% CI)	Heterogeneity measure (I^2^ statistic)	Certainty of evidence (GRADE)
	Intervention	Control				
Change in number of T1 hypointense lesions on cerebral MRI	3057	1884	8	−1.3 (−2.1, −0.5)	93%	⊕⊕⊕⊝
Change in mean volume of T1 hypointense lesions on cerebral MRI	4155	2492	10	−363 (−611, −114)	98%	⊕⊕⊕⊝
Comments	Results of meta‐analyses for both outcomes must be interpreted with caution, as there is considerable heterogeneity in the included studies for both outcomes, which we believe is due to the heterogeneous nature of the interventions of interest.					

Note

Population: adult patients diagnosed with any phenotype of MS based on the McDonald criteria or Definite MS based on the Poser criteria.

Index: FDA approved DMTs, at any dose, frequency, or administration route.

Comparator: placebo, routine care, or no treatment regimen.

Timing: any.

Setting: any.

Mean difference for the volume of lesions is in the unit of mm^3^.

Mean difference for the number of lesions is in the unit of numbers.

Although, these results must be interpreted with caution, as there was considerable heterogeneity in the results of the included studies. Considering this high heterogeneity, the question of why we did statistical quantitative synthesis might rise. The reason is most MS patients undergo a combination therapy of DMTs. Taking this into account, we believe our results give a rough estimate of how much effect, on average, should be expected on T1 hypointense lesions.

Our previous meta‐analysis^12^ revealed that there is a positive correlation between the volume of T1 hypointense lesions and clinical disability measures. One overview of systematic reviews^36^ found that there is good evidence that DMTs improve short‐term (≤2–3 years) disability progression outcomes relative to placebo in at least a subtype of MS patients (relapsing‐remitting MS). Given our current results, it might be hypothesized that one of the mechanisms that result in the reduction of clinical disability progression by DMTs might be due to their effect on reducing T1 hypointense lesions volume (and also probably their frequency). However, this hypothesis requires further investigation. Also, this mechanism cannot be the only one, as we previously^12^ showed that the correlation between lesion load and clinical disability is weak to moderate. To better understand the correlation between cerebral lesions and clinical disability in MS patients, and the effects of DMTs on these outcomes, further studies are required.

### 4.2 Limitations of evidence

The heterogeneity between the results of the included studies was high. We believe one of the main reasons for such heterogeneous results was due to the substantial difference between the drugs in the DMT group and the fact that they act through different mechanisms. This factor also restricted our ability to assess dose–response gradient and publication bias. Overall, although our results give a fundamental perspective on the subject, they are still premature due to the low number of studies (which also restricted our ability to perform subgroup analyses). We confirm that these results are still of limited use for clinical practice, but they provide a good glance of the current state of the knowledge and guide future research to cover the existing gaps.

### 4.3 Limitations of review processes

We encountered a considerable number of studies that probably evaluated the outcome related to our review, but unfortunately, did not report the results. We tried to reach the authors for the data, but were not successful or were not provided with the data. Including those studies could have had a substantial effect on our results. Also, there were not enough studies for each drug to enable us to perform subgroup analyses to assess its effectiveness separately.

### 4.4 Implications

Our results indicate that the use of DMTs, although possibly in various degrees, results in the reduction of frequency and volume T1 hypointense lesions. These findings might help us to gain a better understanding of the possible mechanisms DMTs affect various MS patients’ outcomes. Considering that we found high degrees of heterogeneity between the results of the included studies, future researchers are encouraged to assess the effects of each DMT separately on this outcome as we could not find enough studies for this purpose. We also encourage researchers to report their results more comprehensively, considering that the number of studies we found for this subject is very low compared with the number of trials of DMTs on MS patients, which might have also evaluated this outcome, but did not report it. Another recommendation for future researchers is to assess the effects of DMTs on other MRI outcome measures, such as T2 and gadolinium‐enhancing lesions.

## ADMINISTRATIVE INFORMATION

### Registration

PROSPERO registration ID: CRD42021262883.

### Protocol

The protocol is published elsewhere.^37^

### Amendments

The main difference between the final review and its protocol was that we decided to also assess the change in the number of lesions. There were also some changes of roles in the final review, compared with the roles reported in the protocol. Finally, parts of the methodologies reported in the protocol that was not applicable to the final review were not presented in this manuscript. The rest of the study was conducted according to the study's protocol.

## CONFLICTS OF INTEREST

None.

## AUTHOR CONTRIBUTIONS

AV and MF contributed to coordination of the review, designed and wrote the review, and performed the search. AV, MF, MAS, and ARZ designed the protocol. MaS and MT contributed to study selection and data extraction and assessed the risk of bias in included studies. AV contributed to analysis of data and assessed the confidence in cumulative evidence. AV, MF, and ARZ contributed to interpretation of the results.

## Supporting information

Supplementary Material

Supplementary Material

## Data Availability

All the data that were used in the conduction of this review are publicly available as supplementary files. Not all included studies in this review provided their original data. To access the data of the included studies, we suggest contacting the authors of those studies.
