# Supplementary material for: Disease‐modifying therapies and T1 hypointense lesions in patients with multiple sclerosis: A systematic review and meta‐analysis
Source: CNS Neurosci Ther. 2022 Feb 25;28(5):648–57. doi: 10.1111/cns.13815 (PMC8981477; doi:10.1111/cns.13815)
Supplement: Supplementary file 1 — Supplementary Material [file CNS-28-648-s002.pdf]

Appendix A

Search Strategies

Database and date	Search algorithm

**Embase**<br>Elsevier<br><1994 to 20.03.2021>	#1 'randomized controlled trial'/de<br>#2 'controlled clinical study'/de<br>#3 #1 OR #2<br>#4 random*:ti,ab<br>#5 'randomization'/de<br>#6 'intermethod comparison'/de<br>#7 placebo:ti,ab<br>#8 (compare:ti OR compared:ti OR comparison:ti)<br>#9 ((evaluated:ab OR evaluate:ab OR evaluating:ab OR assessed:ab OR assess:ab) AND<br>(compare:ab OR compared:ab OR comparing:ab OR comparison:ab))<br>#10 (open NEXT/1 label):ti,ab<br>#11 ((double OR single OR doubly OR singly) NEXT/1 (blind OR blinded OR blindly)):ti,ab<br>#12 'double blind procedure'/de<br>#13 (parallel NEXT/1 group*):ti,ab<br>#14 (crossover:ti,ab OR 'cross over':ti,ab)<br>#15 ((assign* OR match OR matched OR allocation) NEAR/6 (alternate OR group OR<br>groups OR intervention OR interventions OR patient OR patients OR subject OR subjects<br>OR participant OR participants)):ti,ab<br>#16 (assigned:ti,ab OR allocated:ti,ab)<br>#17 (controlled NEAR/8 (study OR design OR trial)):ti,ab<br>#18 (volunteer:ti,ab OR volunteers:ti,ab)<br>#19 'human experiment'/de<br>#20 Trial:ti<br>#21 #4 OR #5 OR #6 OR #7 OR #8 OR #9 OR #10 OR #11 OR #12 OR #13 OR #14 OR #15 OR #16 OR #17 OR #18 OR #19 OR #20<br>#22 #21 NOT #3<br>#23 (((random* NEXT/1 sampl* NEAR/8 ('cross section*' OR questionnaire* OR survey OR surveys OR database or databases)):ti,ab) NOT ('comparative study'/de OR<br>'controlled study'/de OR 'randomised controlled':ti,ab OR 'randomized controlled':ti,ab<br>OR 'randomly assigned':ti,ab))<br>#24 ('cross-sectional study'/de NOT ('randomized controlled trial'/de OR 'controlled<br>clinical study'/de OR 'controlled study'/de OR 'randomised controlled':ti,ab OR 'randomized controlled':ti,ab OR 'control group':ti,ab OR 'control groups':ti,ab))<br>#25 ('case control*':ti,ab AND random*:ti,ab NOT ('randomised controlled':ti,ab OR<br>'randomized controlled':ti,ab))<br>#26 ('systematic review':ti NOT (trial:ti OR study:ti))<br>#27 (nonrandom*:ti,ab NOT random*:ti,ab)

#28 ‘random field*’:ti,ab
#29 (‘random cluster’ NEAR/4 sampl*):ti,ab
#30 (review:ab AND review:it NOT trial:ti)
#31 (‘we searched’:ab AND (review:ti,tt OR review:it))
#32 ‘update review’:ab
#33 (databases NEAR/5 searched):ab
#34 ((rat:ti,tt OR rats:ti OR mouse:ti OR mice:ti OR swine:ti OR porcine:ti OR murine:ti
OR sheep:ti OR lambs:ti OR pigs:ti OR piglets:ti OR rabbit:ti OR rabbits:ti OR cat:ti OR
cats:ti OR dog:ti OR dogs: ti OR cattle:ti OR bovine:ti OR monkey:ti OR monkeys:ti OR
trout: ti OR marmoset*:ti) AND ‘animal experiment’/de)
#35 (‘animal experiment’/de NOT (‘human experiment’/de OR ‘human’/de))
#36 #23 OR #24 OR #25 OR #26 OR #27 OR #28 OR #29 OR #30 OR #31 OR #32 OR #33 OR #34 OR #35
#37 #22 NOT #36
#38 'multiple sclerosis'/exp
#39 'multiple sclerosis':ab,ti,kw
#40 #38 OR #39
#41 ('black hole*':ab,ti,kw) OR ('blackhole*':ab,ti,kw)
#42 (hypointens* NEAR/6 t1):ab,ti,kw OR (hypointens* NEAR/6 t1-weighted):ab,ti,kw
OR (hypointens* NEAR/6 t1 weighted):ab,ti,kw OR (hypointens* NEAR/6 t1-w):ab,ti,kw OR (hypointens* NEAR/6 t1w):ab,ti,kw
#43 (t1 NEAR/6 lesion*):ab,ti,kw OR (t1-weighted NEAR/6 lesion*):ab,ti,kw OR (t1 weighted NEAR/6 lesion*):ab,ti,kw OR (t1-w NEAR/6 lesion*):ab,ti,kw OR (t1w NEAR/6 lesion*):ab,ti,kw
#44 #41 OR #42 OR #43
#45 'beta 1a interferon'/exp OR 'beta 1a interferon':ab,ti,kw OR 'interferon beta-1a':ab,ti,kw OR 'interferon beta 1a':ab,ti,kw OR 'interferon beta1a':ab,ti,kw OR avonex:ab,ti,kw OR rebif:ab,ti,kw OR extavia:ab,ti,kw OR betaseron:ab,ti,kw
#46 'peginterferon beta1a'/exp OR 'peginterferon beta1a':ab,ti,kw OR 'peginterferon beta-1a':ab,ti,kw OR 'beta1a peginterferon':ab,ti,kw OR 'beta-1a peginterferon':ab,ti,kw OR
'pegylated interferon beta-1a':ab,ti,kw OR 'pegylated interferon beta1a':ab,ti,kw OR
'pegylated interferon beta 1a':ab,ti,kw OR 'beta-1a pegylated interferon':ab,ti,kw OR
'beta1a pegylated interferon':ab,ti,kw OR 'beta 1a pegylated interferon':ab,ti,kw OR
plegridy:ab,ti,kw
#47 'glatiramer'/exp OR glatiramer:ab,ti,kw OR copaxone:ab,ti,kw OR glatopa:ab,ti,kw

#48 'fumaric acid dimethyl ester'/exp OR 'fumaric acid dimethyl ester':ab,ti,kw OR 'dimethyl fumarate':ab,ti,kw OR tecfidera:ab,ti,kw
#49 'teriflunomide'/exp OR teriflunomide:ab,ti,kw OR aubagio:ab,ti,kw
#50 'fingolimod'/exp OR fingolimod:ab,ti,kw OR gilenia:ab,ti,kw OR gilenya:ab,ti,kw
#51 'siponimod'/exp OR siponimod:ab,ti,kw OR mayzent:ab,ti,kw
#52 'ozanimod'/exp OR ozanimod:ab,ti,kw OR zeposia:ab,ti,kw
#53 'natalizumab'/exp OR natalizumab:ab,ti,kw OR tysabri:ab,ti,kw
#54 'alemtuzumab'/exp OR alemtuzumab:ab,ti,kw OR lemtrada:ab,ti,kw
#55 'ocrelizumab'/exp OR ocrelizumab:ab,ti,kw OR ocrevus:ab,ti,kw
#56 'rituximab'/exp OR rituximab:ab,ti,kw OR mabthera:ab,ti,kw
#57 'mitoxantrone'/exp OR mitoxantrone:ab,ti,kw OR novantrone:ab,ti,kw
#58 'cladribine'/exp OR cladribine:ab,ti,kw OR mavenclad:ab,ti,kw
#59 'ofatumumab'/exp OR ofatumumab:ab,ti,kw OR arzerra:ab,ti,kw
#60 'daclizumab'/exp OR daclizumab:ab,ti,kw OR zinbryta:ab,ti,kw
#61 'diroximel fumarate'/exp OR 'diroximel fumarate':ab,ti,kw OR vumerity:ab,ti,kw
#62 'disease modifying therap*':ab,ti,kw OR 'disease-modifying therap*':ab,ti,kw OR
dmt*:ab,ti,kw
#63 #45 OR #46 OR #47 OR #48 OR #49 OR #50 OR #51 OR #52 OR #53 OR #54 OR #55 OR #56 OR #57 OR #58 OR #59 OR #60 OR #61 OR #62
#64 #37 AND #40 AND #44 AND #63

MEDLINE

Ovid

<1946 to 20.03.2021>

1 randomized controlled trial.pt.
2 controlled clinical trial.pt.
3 randomized.ab.
4 placebo.ab.
5 clinical trials as topic.sh.
6 randomly.ab.
7 trial.ti.
8 1 or 2 or 3 or 4 or 5 or 6 or 7
9 exp animals/ not humans.sh.
10 8 not 9
11 multiple sclerosis.mp.
12 (black hole* or blackhole*).mp.
13 ((hypointens* adj6 t1) or (hypointens* adj6 t1-weighted) or (hypointens* adj6 t1
weighted) or (hypointens* adj6 t1-w) or (hypointens* adj6 t1w)).mp.
14 ((t1 adj6 lesion*) or (t1-weighted adj6 lesion*) or (t1 weighted adj6 lesion*) or (t1-w
adj6 lesion*) or (t1w adj6 lesion*)).mp.
15 12 or 13 or 14
16 (Interferon beta-1a or beta 1a interferon or beta1a interferon or interferon beta 1a or
interferon beta1a or avonex or rebif or extavia or betaseron).mp.
17 (peginterferon beta1a or peginterferon beta-1a or beta1a peginterferon or beta-1a
peginterferon or pegylated interferon beta-1a or pegylated interferon beta1a or pegylated
interferon beta 1a or beta-1a pegylated interferon or beta1a pegylated interferon or beta
1a pegylated interferon or plegridy).mp.
18 (Glatiramer Acetate or glatiramer or copaxone or glatopa).mp.
19 (Dimethyl Fumarate or fumaric acid dimethyl ester or tecfidera).mp.
20 (teriflunomide or aubagio).mp.
21 (Fingolimod Hydrochloride or fingolimod or gilenia or gilenya).mp.
22 (siponimod or mayzent).mp.
23 (ozanimod or zeposia).mp.
24 (Natalizumab or tysabri).mp.
25 (Alemtuzumab or lemtrada).mp.
26 (ocrelizumab or ocrevus).mp.
27 (Rituximab or mabthera).mp.
28 (Mitoxantrone or novantrone).mp.
29 (Cladribine or mavenclad).mp.
30 (ofatumumab or arzerra).mp.
31 (Daclizumab or zinbryta).mp.
32 (diroximel fumarate or vumerity).mp.
33 (disease modifying therap* or disease-modifying therap* or dmt*).mp.

	34 16 or 17 or 18 or 19 or 20 or 21 or 22 or 23 or 24 or 25 or 26 or 27 or 28 or 29 or 30 or<br>31 or 32 or 33<br>35 10 and 11 and 15 and 34

**The Cochrane Library**<br>10.02.2021	#1 MeSH descriptor: [Multiple Sclerosis] explode all trees<br>#2 (multiple sclerosis):ti,ab,kw<br>#3 #1 OR #2<br>#4 (black hole*):ti,ab,kw OR (blackhole*):ti,ab,kw<br>#5 ((hypointens* NEAR/6 t1) OR (hypointens* NEAR/6 t1 weighted) OR (hypointens*<br>NEAR/6 t1w)):ti,ab,kw<br>#6 ((t1 NEAR/6 lesion*) OR (t1 weighted NEAR/6 lesion*) OR (t1w NEAR/6<br>lesion*)):ti,ab,kw<br>#7 #4 OR #5 OR #6<br>#8 MeSH descriptor: [Interferon beta-1a] explode all trees<br>#9 (Interferon beta-1a OR beta 1a interferon OR beta1a interferon OR interferon beta 1a<br>OR interferon beta1a OR avonex OR rebif OR extavia OR betaseron):ti,ab,kw<br>#10 (peginterferon beta1a OR peginterferon beta-1a OR beta1a peginterferon OR beta-1a<br>peginterferon OR pegylated interferon beta-1a OR pegylated interferon beta1a OR pegylated interferon beta 1a OR beta-1a pegylated interferon OR beta1a pegylated<br>interferon OR beta 1a pegylated interferon OR plegridy):ti,ab,kw<br>#11 MeSH descriptor: [Glatiramer Acetate] explode all trees<br>#12 (glatiramer OR copaxone OR glatopa):ti,ab,kw<br>#13 MeSH descriptor: [Dimethyl Fumarate] explode all trees<br>#14 (dimethyl fumarate OR fumaric acid dimethyl ester OR tecfidera):ti,ab,kw<br>#15 (teriflunomide OR aubagio):ti,ab,kw<br>#16 MeSH descriptor: [Fingolimod Hydrochloride] explode all trees<br>#17 (fingolimod OR gilenia OR gilenya):ti,ab,kw<br>#18 (siponimod OR mayzent):ti,ab,kw<br>#19 (ozanimod OR zeposia):ti,ab,kw<br>#20 MeSH descriptor: [Natalizumab] explode all trees<br>#21 (natalizumab OR tysabri):ti,ab,kw<br>#22 MeSH descriptor: [Alemtuzumab] explode all trees<br>#23 (alemtuzumab OR lemtrada):ti,ab,kw<br>#24 (ocrelizumab OR ocrevus):ti,ab,kw<br>#25 MeSH descriptor: [Rituximab] explode all trees<br>#26 (rituximab OR mabthera):ti,ab,kw<br>#27 MeSH descriptor: [Mitoxantrone] explode all trees<br>#28 (mitoxantrone OR novantrone):ti,ab,kw<br>#29 MeSH descriptor: [Cladribine] explode all trees<br>#30 (cladribine OR mavenclad):ti,ab,kw<br>#31 (ofatumumab OR arzerra):ti,ab,kw<br>#32 MeSH descriptor: [Daclizumab] explode all trees<br>#33 (daclizumab or zinbryta):ti,ab,kw<br>#34 (diroximel fumarate OR vumerity):ti,ab,kw<br>#35 (disease modifying therap* OR dmt*):ti,ab,kw

	#36 #8 OR #9 OR #10 OR #11 OR #12 OR #13 OR #14 OR #15 OR #16 OR #17 OR #18 OR #19 OR #20 OR #21 OR #22 OR #23 OR #24 OR #25 OR #26 OR #27 OR #28 OR #29 OR #30 OR #31 OR #32 OR #33 OR #34 OR #35
	#37 #3 AND #7 AND #36
