# Supplementary material for: Disease‐modifying therapies and T1 hypointense lesions in patients with multiple sclerosis: A systematic review and meta‐analysis
Source: CNS Neurosci Ther. 2022 Feb 25;28(5):648–57. doi: 10.1111/cns.13815 (PMC8981477; doi:10.1111/cns.13815)
Supplement: Supplementary file 2 — Supplementary Material [file CNS-28-648-s001.pdf]

Appendix B

The RoB2 tool and its variants

Signaling questions	Authors' judgment for 'yes'
**Domain 1: Risk of bias arising from the randomization process**	
1.1 Was the allocation sequence random?	The use of randomization methods is clearly stated in the study.
1.2 Was the allocation sequence concealed until participants were enrolled and assigned to interventions?	Allocation sequence concealment is clearly stated.
1.3 Did baseline differences between intervention groups suggest a problem with the randomization process?	Age, sex, and ethnicityare not significantly different between the groups.
**Domain 1b: Risk of bias arising from the timing of identification or recruitment of participants in a cluster-randomized trial (for Cluster randomized controlled trials only)**	
1b.1 Were all the individual participants identified and recruited (if appropriate) before the randomization of clusters?	All participants were identified and recruited before the clusters were randomized, or individual participants were not recruited at all but all were identified before randomization.
1b.2 If N/PN/NI to 1b.1: Is it likely that the selection of individual participants was affected by knowledge of the intervention assigned to the cluster?	Recruiting individuals were aware of cluster allocation before recruitment, or some participants were aware of cluster allocation before their recruitment, or those identifying potential participants (when recruitment is to take place subsequently) are aware of cluster allocation, or those identifying actual participants (when there is no subsequent recruitment) are aware of cluster allocation.
1b.3 Were there baseline imbalances that suggest differential identification or recruitment of individual participants between intervention groups?	Imbalances that are compatible with a chance should not be interpreted as suggesting differential identification or recruitment of participants.
**Domain S: Risk of bias arising from period and carryover effects (for Cross-over randomized controlled trials only)**	

S.1 Was the number of participants allocated to each of the two sequences equal or nearly equal?	No: We only include the results from the first phase of a cross-over trial.
S.2 If N/PN/NI to S.1: Were period effects accounted for in the analysis?	No: We only include the results from the first phase of a cross-over trial.
S.3 Was there sufficient time for any carryover effects to have disappeared before outcome assessment in the second period?	No: We only include the results from the first phase of a cross-over trial.
**Domain 2: Risk of bias due to deviations from the intended interventions (effect of assignment to intervention)**	
2.1. Were participants aware of their assigned intervention during the trial?	The allocation sequence was concealed for patients.
2.2. Were carers and people delivering the interventions aware of participants' assigned interventions during the trial?	The allocation sequence was concealed for care providers.
2.3. If Y/PY/NI to 2.1 or 2.2: Were there deviations from the intended intervention that arose because of the trial context?	There is evidence or strong reason to believe that the trial context led to failure to implement the protocol interventions or to implementation of interventions not allowed by the protocol.
2.4 If Y/PY to 2.3: Were these deviations likely to have affected the outcome?	Deviations are likely to affect the outcome.
2.5. If Y/PY/NI to 2.4: Were these deviations from intended intervention balanced between groups?	Deviations are not balanced between the intervention groups.
2.6 Was an appropriate analysis used to estimate the effect of assignment to intervention?	Intention-to-treat (ITT) analyses and modified intention-to-treat (mITT) analyses are appropriate.

2.7 If N/PN/NI to 2.6: Was there potential for a substantial impact (on the result) of the failure to analyze participants in the group to which they were randomized?	The number of participants who were analyzed in the wrong intervention group, or excluded from the analysis, was sufficient that there could have been a substantial impact on the result.
**Domain 2: Risk of bias due to deviations from the intended interventions (effect of adhering to intervention)**	
2.1. Were participants aware of their assigned intervention during the trial?	Participants were not masked.
2.2. Were carers and people delivering the interventions aware of participants' assigned interventions during the trial?	Care providers were not masked.
2.3. [If applicable:] If Y/PY/NI to 2.1 or 2.2: Were important non-protocol interventions balanced across intervention groups?	Important non-protocol interventions are the additional interventions or exposures that: (1) are inconsistent with the trial protocol; (2) trial participants might receive with or after starting their assigned intervention; and (3) are prognostic for the outcome.
2.4. [If applicable:] Were there failures in implementing the intervention that could have affected the outcome?	The intervention was not implemented as intended.
2.5. [If applicable:] Was there non-adherence to the assigned intervention regimen that could have affected participants' outcomes?	Non-adherence includes imperfect compliance with a sustained intervention, cessation of intervention, crossovers to the comparator intervention, and switches to another active intervention.
2.6. If N/PN/NI to 2.3, or Y/PY/NI to 2.4 or 2.5: Was an appropriate analysis used to estimate the effect of adhering to the intervention?	Appropriate methods include (1) instrumental variable analyses to estimate the effect of receiving the assigned intervention in trials in which a single intervention, administered only at baseline and with all-or-nothing adherence, is compared with standard care; and (2) inverse probability weighting to adjust for censoring of participants who cease adherence to their assigned intervention, in trials of sustained treatment strategies.
**Domain 3: Risk of bias due to missing outcome data**	

3.1 Were data for this outcome available for all, or nearly all, participants randomized?	Availability of data from 95% of the participants will be considered sufficient (imputed data will be regarded as missing data).
3.2 If N/PN/NI to 3.1: Is there evidence that the result was not biased by missing outcome data?	Analysis methods corrected for bias, or sensitivity analyses were performed showing that results are little changed under a range of plausible assumptions about the relationship between missingness in the outcome and its true value.
3.3 If N/PN to 3.2: Could missingness in the outcome depend on its true value?	Loss to follow-up, or withdrawal from the study, could be related to participants' health status.
3.4 If Y/PY/NI to 3.3: Is it likely that missingness in the outcome depended on its true value?	There are differences between intervention groups in the proportions of missing outcome data. Reported reasons for missing outcome data differ between the intervention groups; or reported reasons for missing outcome data to provide evidence that missingness in the outcome depends on its true value.
**Domain 4: Risk of bias in the measurement of the outcome**	
4.1 Was the method of measuring the outcome inappropriate?	No: cerebral MRI is the only available method for measuring T1 hypointense lesions.
4.2 Could measurement or ascertainment of the outcome have differed between intervention groups?	No: cerebral MRI is the only available method for measuring T1 hypointense lesions.
4.3 If N/PN/NI to 4.1 and 4.2: Were outcome assessors aware of the intervention received by study participants?	Outcome assessors were not masked.
4.4 If Y/PY/NI to 4.3: Could assessment of the outcome have been influenced by knowledge of intervention received?	No: our outcome of interest is not susceptible to the judgment of the assessors.
4.5 If Y/PY/NI to 4.4: Is it likely that assessment of the outcome was influenced by knowledge of intervention received?	No: our outcome of interest is not susceptible to the judgment of the assessors.
**Domain 5: Risk of bias in the selection of the reported result**	

5.1 Were the data that produced this result analyzed following a pre-specified analysis plan that was finalized before unblinded outcome data were available for analysis?	Finalization of the analysis intentions precedes the availability of unblinded outcome data to the trial investigators.
Is the numerical result being assessed likely to have been selected, based on the results, from...	-
5.2. ... multiple eligible outcome measurements (e.g. scales, definitions, time points) within the outcome domain?	No: the volume of cerebral T1 hypointense lesion can only be measured on one scale.
5.3 ... multiple eligible analyses of the data?	There is strong evidence that the numerical results have been selected from multiple eligible analyses of the data (e.g. unadjusted and adjusted models; final value vs change from baseline vs analysis of covariance).
5.4 Is a result based on data from both periods sought, but unavailable based on carryover having been identified? (for Cross-over trials only)	No: We include only the results of the first phase of a crossover trial.
Y: Yes; PY: Probably Yes; PN: Probably No; N: No; NI: No Information	
