# Supplementary material for: Disease‐modifying therapies and T1 hypointense lesions in patients with multiple sclerosis: A systematic review and meta‐analysis
Source: CNS Neurosci Ther. 2022 Feb 25;28(5):648–57. doi: 10.1111/cns.13815 (PMC8981477; doi:10.1111/cns.13815)
Supplement: Supplementary file 3 [file CNS-28-648-s003.docx]

| Population: adult patients diagnosed with any phenotype of MS based on the McDonald criteria or Definite MS based on the Poser criteria.  Index: FDA approved DMTs, at any dose, frequency, or administration route.  Comparator: placebo, routine care, or no treatment regimen.  Timing: any.  Setting: any. | | | | | | |
| --- | --- | --- | --- | --- | --- | --- |
| **Outcome** | **Number of participants** | | **Number of studies** | **Mean difference (95% CI)** | **Heterogeneity measure (I^2^ statistic)** | **Certainty of evidence (GRADE)** |
|  | **Intervention** | **Control** |  |  |  |  |
| Change in number of T1 hypointense lesions on cerebral MRI | 3057 | 1884 | 8 | -1.3 (-2.1, -0.5) | 93% | ⊕⊕⊕⊝ |
| Change in mean volume of T1 hypointense lesions on cerebral MRI | 4155 | 2492 | 10 | -363 (-611, -114) | 98% | ⊕⊕⊕⊝ |
| **Comments** | Results of meta-analyses for both outcomes must be interpreted with caution, as there is considerable heterogeneity in the included studies for both outcomes, which we believe is due to the heterogeneous nature of the interventions of interest. | | | | | |
| Mean difference for the volume of lesions is in the unit of millimeters^3^.  Mean difference for the number of lesions is in the unit of numbers. | | | | | | |
